# Case Report: Tumor regression and neurological recovery in paraplegia from POLD1-mutated hepatocellular carcinoma treated with targeted immunotherapy and electroacupuncture

**DOI:** 10.3389/fimmu.2026.1776269

**Published:** 2026-06-01

**Authors:** Xionghao Pang, Xinyi Zhao, Jiajian Lin, Yansu Wang, Liang Xiao, Meixiang Li, Lin Dong, Yaoguang Xie, Lingzhi Jiang, Caiping Yang, Geng Tian

**Affiliations:** 1 Department of Oncology, The First Affiliated Hospital of Shenzhen University, Health Science Center, Shenzhen Second People’s Hospital, Shenzhen, China; 2 Shenzhen Xiao Chuanguo Hospital, Shenzhen, China; 3 Department of Pain Management and Rehabilitation, Nan’ao People’s Hospital of Dapeng New District, Shenzhen, China

**Keywords:** complete remission, electroacupuncture, germline mutation, hepatocellular carcinoma, immunotherapy, POLD1, spinal metastasis

## Abstract

**Background:**

Hepatocellular carcinoma (HCC) patients with bone metastasis-induced paraplegia have an extremely poor prognosis. The potential for neurological recovery following immunotherapy and subsequent rehabilitation in this population remains unclear.

**Case:**

A 58-year-old male with Barcelona Clinic Liver Cancer stage C (BCLC) HCC and T11 vertebral metastasis causing American Spinal Injury Association (ASIA) grade C paraplegia carried a rare POLD1 c.1745C>T (p.T582M) germline mutation with low tumor mutational burden (TMB) and microsatellite instability-low status (MSI-L, blood-based MSI-L (bMSI-L) 7.76%). Immunotherapy with atezolizumab (1200 mg every 3 weeks [Q3W]) combined with targeted therapy with bevacizumab (600 mg Q3W) was initiated in July 2021.A substantial temporal gap of approximately 21 months followed, after which electroacupuncture-based rehabilitation (5-Hz continuous-wave, stimulating Jiaji acupoints, Zusanli, etc) was started in May 2023.

**Results:**

Following three months of targeted immunotherapy, the tumor exhibited complete response (CR) according to modified Response Evaluation Criteria in Solid Tumors (mRECIST). At the 28-month follow-up assessment, the patient was eventually able to walk independently with a walker. Serial cytokine measurements revealed two distinct peaks in IL-1β, TNF-α, and IFN-γ relative to baseline (2021-06-25): an initial rise after immunotherapy initiation (IL-1β up 13.7-fold; TNF-α up 2.5-fold; IFN-γ up 33.3-fold) and a second, larger increase during electroacupuncture rehabilitation coinciding with neurological recovery (IL-1β up 31.8-fold; TNF-α up 7.2-fold; IFN-γ up 96.1-fold). These observations suggest temporal associations between immune activation and clinical milestones, but causality cannot be established from a single case.

**Conclusion:**

This case suggests that POLD1 mutation, despite low TMB and MSI-L, may be associated with enhanced sensitivity to immunotherapy in HCC, though the underlying mechanisms require further investigation. Targeted immunotherapy combined with electroacupuncture may influence neural remodeling by modulating a pro-reparative inflammatory microenvironment, which could potentially support functional reconstruction in patients with spinal metastases. However, there is no direct evidence that immunotherapy accelerates neural recovery, and these observations should be interpreted as hypothesis-generating findings requiring rigorous testing in prospective studies.

## 1 Introduction

### 1.1 Disease background

Hepatocellular carcinoma(HCC)is the most common type of primary malignant liver tumor, demonstrating highly malignant biological behavior and a tendency to develop intrahepatic and extrahepatic metastases. Bone is the third most common site of extrahepatic metastasis in HCC (after the lungs and lymph nodes) (1). After bone metastasis occurs, HCC patients’ quality of life and the effects of treatment are significantly reduced. In Asia, hepatitis B virus (HBV) infections are common in HCC patients and may be associated with an increased incidence of bone metastasis and bone-related events (2). However, early symptoms of HCC bone metastasis are often atypical, leading to delayed diagnosis and poor prognosis. While conventional treatments for HCC, such as surgical resection, liver transplantation, radiofrequency ablation, and transarterial chemoembolization (TACE) (1), have achieved noteworthy results, the rapid development of targeted therapy and immunotherapy have provided more new treatment options to patients with advanced liver cancer, particularly those with bone metastasis.

### 1.2 Research gap

Despite its prevalence among this patient population, rehabilitation and functional improvement related to nerve injury are rarely reported within clinical practice. Recent studies have shown that abnormalities in DNA damage repair genes (such as POLD1) may enhance immunogenicity by increasing the expression of tumor neoantigens (3), but there is currently a lack of clinical evidence elucidating their role in HCC bone metastasis.

### 1.3 Research objectives

This paper analyzes a case of HCC bone metastasis with complete remission and significant neurological function recovery to explore the potential mechanisms involved in comprehensive treatment plans, as well as their clinical significance.

## 2 Case presentation

### 2.1 Baseline characteristics, initial diagnosis, and treatment

**Figure 1:**
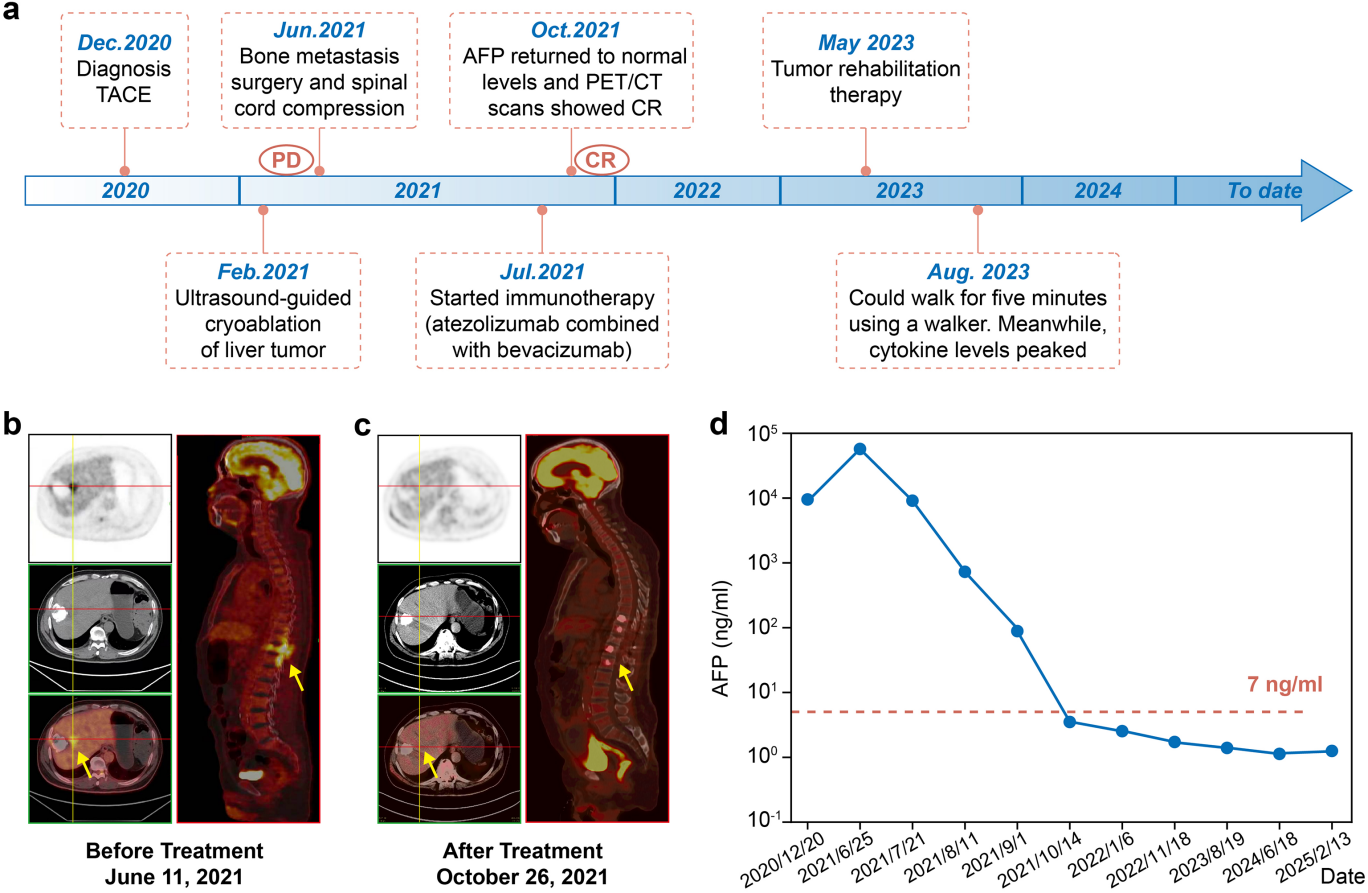
**(a)** Timeline showing key clinical events from diagnosis to rehabilitation. **(b)** Pre-immunotherapy PET/CT images (June 2021) showing liver tumor and bone metastases (yellow arrows). **(c)** Post-immunotherapy images (October 2021) demonstrating complete response. **(d)** AFP levels over time, showing decline and sustained normalization at 40-month follow-up.

A 58-year-old male with a 30-year history of chronic hepatitis B presented with persistent right upper abdominal pain in December 2020. Laboratory tests showed an alpha-fetoprotein (AFP) level of 8406 ng/mL, and imaging confirmed HCC(Barcelona Clinic Liver Cancer [BCLC] stage C) with metastasis to the hepatic hilum and retroperitoneal lymph nodes. The patient declined surgery and received transarterial chemoembolization (TACE) and multiple ablations (cryoablation/microwave ablation), but the disease continued to progress. In June 2021, the patient developed lower limb weakness and urinary retention. Imaging confirmed multiple bone metastases in the thoracolumbar spine and pathological fracture of the T11 vertebral body with spinal cord compression. After emergency surgery, severe neurological deficits remained, with American Spinal Injury Association (ASIA) grade C paraplegia and lower extremity muscle strength of grade 0–2. Combination immunotherapy and targeted therapy with atezolizumab (1200 mg every 3 weeks [Q3W]) plus bevacizumab (600 mg Q3W) was initiated in July 2021. His whole disease course and clinical outcomes were summarized in **Figure 1a**.

Imaging: Positron emission tomography/computed tomography (PET/CT) scans performed in October 2021 showed complete metabolic inhibition of the tumor (maximum standardized uptake value [SUVmax] decreased from 6.5 to 2.5), achieving Modified Response Evaluation Criteria in Solid Tumors (mRECIST) (4) for complete response (CR) (**Figures 1b, c**).

Tumor markers: After 1 cycle, AFP levels decreased from 60500 ng/mL to 9201 ng/mL (↓84.79%), and returned to normal in October 2021, where it remained (**Figure 1d**).

### 2.2 Electroacupuncture rehabilitation and neurological function reconstruction

Beginning in May 2023, the patient received comprehensive rehabilitation primarily in the form of electroacupuncture (Jiaji acupoints, Zusanli, and others; 5Hz continuous wave, 30 minutes daily).

Motor function: His ASIA motor score increased from a baseline of 58 points (grade C) to 67 points (grade D) at 12 months. Hip/knee muscle strength recovered from grade 2 to grade 4, and the patient could walk independently with a walker for ≥50 meters.

**Figure 2:**
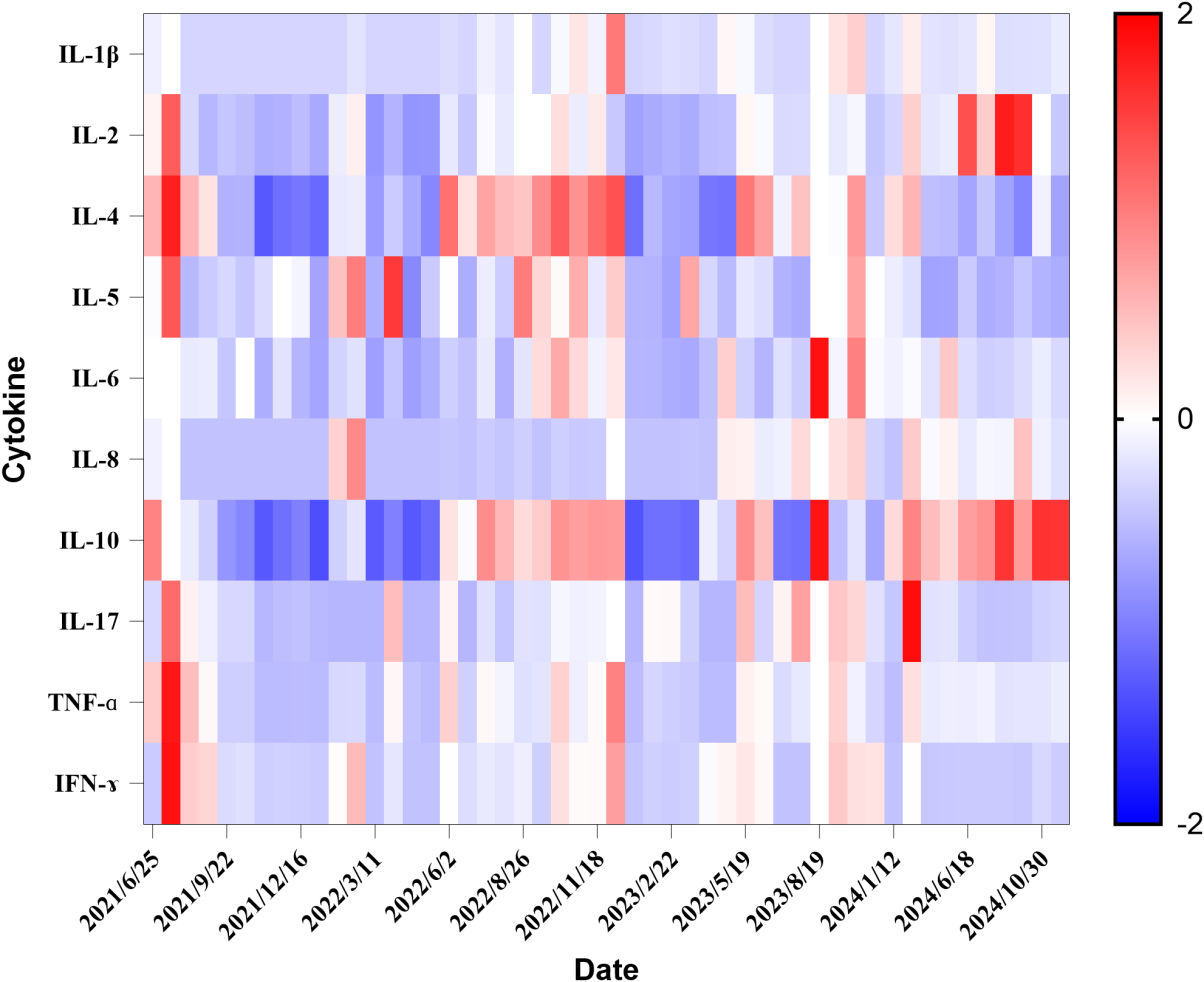
Heatmap showing temporal changes in cytokine levels during treatment and follow-up (2021–2024). Each row represents a cytokine, and each column represents a sampling time point. Colors indicate relative expression levels after row-wise Z-score normalization: red represents expression above the mean (Z-score > 0), blue represents expression below the mean (Z-score < 0), and white represents mean expression (Z-score = 0). The color scale ranges from–2 to 2.

Immune-neural correlation: After 3 months of rehabilitation, IL-1β, TNF-α, and IFN-γ increased 31.8-fold, 7.2-fold, and 96.1-fold of the baseline, (**Figure 2**) respectively, in sync with functional recovery (see **Supplementary Data Sheet 2**).

**Figure 3:**
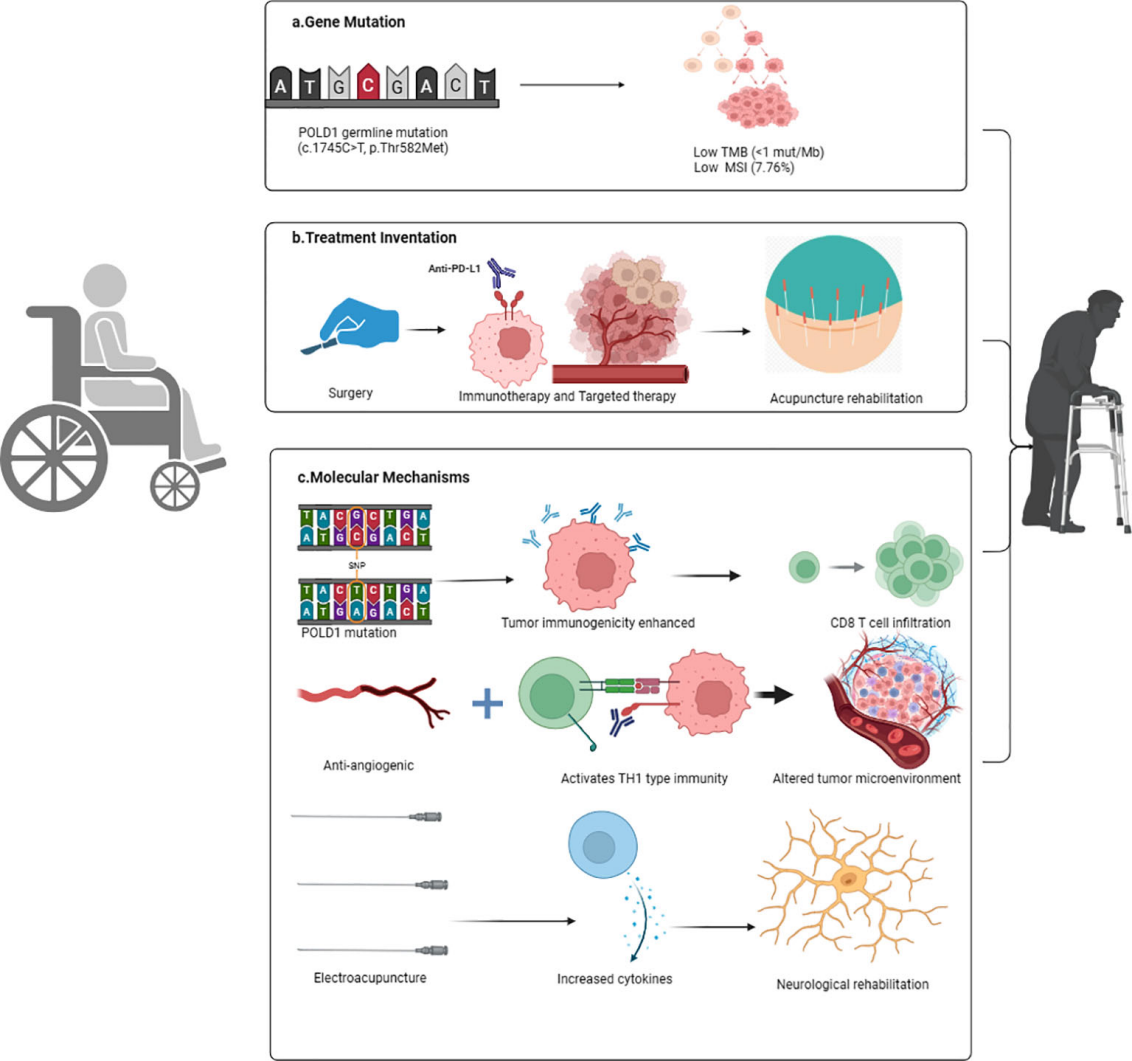
chematic representation of tumor rehabilitation therapy. **(a)** Gene mutation profileshowing the POLD1 mutation and its downstream effects. **(b)** Treatment implementationshowing surgery, immunotherapy/targeted therapy, and acupuncture rehabilitation. **(c)** Molecular mechanisms highlighting immune activation and neurological recovery pathways. Created with BioRender.com.

From initiation of the immunotherapy to date, the patient has experienced durable survivalbenefit of more than 4 years, and he had a good performance status when this case report was finished. The patient's functional recovery is shown in **Supplementary Video 1**. The overall mechanism of tumor rehabilitation therapy is summarized in **Figure 3**.

## 3 Discussion

### 3.1 Clinical uniqueness and breakthrough

Rarity and prognostic breakthrough: We report a rare case of a paraplegic patient with HCC bone metastasis to achieve CR, recover neurological function through multidisciplinary treatment, survive over 50 months, and regain the ability to walk with a walker.

Molecular characteristics: The rare POLD1 c.1745C>T germline mutation (non-catalytic domain, low TMB/MSI) may provide a new target for precise application of immunotherapy.

Mechanistic insights: Notably, changes in the patient’s cytokine profile-–particularly elevations of IL-1β, TNF-α, and IFN-γ during the rehabilitation phase-–temporally coincided with neurological function recovery, suggesting a potential temporal association that may reflect immuno-neural interaction and merits further investigation.

### 3.2 Molecular mechanism analysis

The POLD1 c.1745C>T (p.T582M) mutation identified in this patient is located in the flank of the exonuclease domain (5). Interestingly, unlike canonical exo-domain mutations typically associated with hypermutator phenotypes (6), this patient exhibited low TMB and MSI-L—features typically associated with limited responses to immune checkpoint inhibitors. This apparent discrepancy raises questions about the relationship between POLD1 mutations and TMB, and highlights the need for further investigation into the mechanisms underlying the observed complete response. Several non-mutually exclusive mechanisms may explain this phenomenon. First, POLD1 mutations may influence immunotherapy sensitivity through TMB-independent pathways, such as altering protein stability or modulating the tumor immune microenvironment (7). Second, emerging evidence suggests that neoantigen quality—reflected by their dissimilarity to self-proteins—rather than quantity may be a critical determinant of immunogenicity, even in low-TMB tumors (8). Third, the combination of atezolizumab and bevacizumab may have synergistic effects that are particularly relevant in low-TMB contexts, as vascular normalization can enhance T cell infiltration and functionality (9, 10). Further mechanistic discussion, including the role of neoantigen quality and vascular remodeling, is provided in **Supplementary Material 1**.

Key findings: This case illustrates that durable response to immunotherapy can occur in patients with low TMB and MSI. **This** suggests that factors beyond conventional biomarkers-–such as specific gene mutations (eg POLD1), treatment combination strategies, and host immune status-–may influence clinical outcomes. Comprehensive evaluation of these factors may help refine patient selection for immunotherapy.

### 3.3 Mechanisms behind neurological function recovery

Temporal analysis of cytokine responses: Our serial cytokine monitoring revealed two distinct phases of cytokine elevation. The initial cytokine elevation following immunotherapy initiation (IL-1β up 13.7-fold; TNF-α up 2.5-fold; IFN-γ up 33.3-fold) likely represents the expected systemic immune response to checkpoint inhibition. The second, more pronounced cytokine surge occurred approximately 3 months after the start of electroacupuncture rehabilitation, about 25 months after immunotherapy initiation (IL-1β up 31.8-fold; TNF-α up 7.2-fold; IFN-γ up 96.1-fold), making it unlikely to be a delayed response to the initial immunotherapy. Instead, this temporal alignment with electroacupuncture initiation and neurological recovery suggests that the rehabilitation intervention may have triggered a localized immune activation that potentially facilitated neural repair processes. However, we acknowledge that the causal relationship cannot be definitively established from this single case observation, and the possibility of coincidental timing cannot be excluded.

Dual regulation of electroacupuncture: Local neural repair: Low-frequency (5Hz) electrical stimulation of Jiaji acupoints/Zusanli accelerates motor nerve conduction (electromyography shows increased velocity) and promotes axonal regeneration (11, 12).

Systemic immune regulation: Inhibits pro-inflammatory cytokines such as IL-6 through the vagus nerve-cholinergic pathway, upregulates IFN-γ/TNF-α, forms a “controlled inflammatory microenvironment,” that may support neural regeneration (13). In this patient, the observed cytokine increases during the rehabilitation phase temporally coincided with neurological function recovery. While this temporal association is intriguing and generates the hypothesis of a pro-reparative inflammatory microenvironment, direct mechanistic evidence cannot be established from a single case and requires further investigation.

Evidence of functional improvement: The patient’s ASIA motor score increased from 58 to 67, his muscle strength went from grade 2 to grade 4, and balance/gait/daily activities all saw significant improvement.

### 3.4 Clinical insights

Treatment considerations: This case illustrates the potential benefit of a multidisciplinary approach integrating molecular profiling (e.g, POLD1 mutation) with sequenced interventions—initial immunotherapy for tumor control followed by electroacupuncture-based rehabilitation for neurological recovery. However, this strategy requires validation in larger studies.

Prognostic evaluation: Use tumor molecular characteristics, initial neurological functioning status, and dynamic cytokine monitoring (as measured by the IFN-γ/IL-6 ratio) to predict repair potential.

Future directions: Conduct prospective cohort studies to verify the mechanisms behind electroacupuncture, explore the optimal timing for such interventions, and develop immunotherapy prediction models for low-TMB patients.

### 3.5 Limitations and prospects

Limitations: A single case cannot exclude individual differences; multimodal treatment makes it difficult to distinguish the contributions of the respective therapies; there is a lack of continuous imaging and dynamic cytokine monitoring. In addition, without comprehensive genomic analysis of the tumor and detailed immune microenvironment profiling, the precise role of the POLD1 mutation in treatment response remains speculative. The hypotheses generated here require validation in larger cohorts with systematic molecular characterization. Future directions: Conduct multicenter studies, design crossover trials to separate the effects of immunotherapy and rehabilitation, and analyze immuno-neural interactions using spatial transcriptomics.

## 4 Conclusion

This case suggests that HCC patients with POLD1 mutations may benefit from immunotherapy combined with electroacupuncture, achieving tumor control and recovering neurological function accompanied by changes in the cytokine network that paralleled with functional improvement. This case suggests that integrating precision molecular typing with multidisciplinary rehabilitation—in this instance, immunotherapy followed by electroacupuncture—may offer a new approach for advanced HCC patients with bone metastasis. These observations underscore the value of further investigating immune-neural interactions to potentially improve both tumor control and functional outcomes in this challenging population.

### 4.1 Patient perspective

I am a 58-year-old man. I first experienced pain in the upper right abdomen and was diagnosed with liver cancer. Surgery was recommended, but I chose other treatments such as chemotherapy and ablation. Despite these efforts, my condition worsened. By June 2021, I developed weakness in my legs and could not urinate normally. Tests showed bone metastases in the spine, causing spinal compression and severe mobility loss. After surgery, I became paraplegic and depended on others for movement. Targeted immunotherapy combined with other medications was then given, and my condition gradually improved with a significant reduction in cancer cells. I later tried electroacupuncture; each session brought relief, and I slowly regained leg strength. I can now walk independently with a walker. This experience gave me renewed hope for a better life.

## Data Availability

The raw data supporting the conclusions of this article will be made available by the authors, without undue reservation.

## Supplementary material

Supplementary FILE 1 Extended materials and methods and additional supporting information related to molecular characteristics, immunotherapy response, neurological recovery, and rehabilitation protocol.

Supplementary Data Sheet S2 Raw data supporting the clinical assessments and biomarker analysis.

Supplementary Video S1 Video documentation of the patient's functional recovery, showing the ability to walk independently with a walker after rehabilitation.
