# Supplementary material for: Case Report: Tumor regression and neurological recovery in paraplegia from POLD1-mutated hepatocellular carcinoma treated with targeted immunotherapy and electroacupuncture
Source: Front Immunol. 2026 Jun 1;17:1776269. doi: 10.3389/fimmu.2026.1776269 (PMC13265552; doi:10.3389/fimmu.2026.1776269)
Supplement: Supplementary FILE 1 — Extended materials and methods and additional supporting information related to molecular characteristics, immunotherapy response, neurological recovery, and rehabilitation protocol. [file DataSheet1.pdf]

Additional file

S1. Extended Materials and Methods 1
1.1Molecular Characteristics & Functional Implications of POLD1 Mutation 1
1.2 Immunotherapeutic Responsiveness in Low-TMB Context 2
S2. Multidimensional Mechanisms of Neurological Recovery 3
2.1 Critical Role of Acupuncture in Neural Restoration 3
2.2 Temporal Correlation Between Cytokine Dynamics & Functional Recovery 4
S3.Integrated Rehabilitation and Neurological Recovery (May 2023-Present) 5
Reference 7

S1. Extended Materials and Methods

1.1Molecular Characteristics & Functional Implications of POLD1 Mutation

Longitudinal genomic profiling via targeted NGS revealed a rare germline POLD1 mutation (c.1745C>T, p.Thr582Met) located in the non-catalytic flanking region of the exonuclease domain. Unlike canonical exo-domain mutations typically associated with hypermutator phenotypes, this variant coexisted with low blood tumor mutational burden (bTMB <1 mut/Mb) and minimal microsatellite instability (bMSI-L, 7.76%)[1]. Absence in gnomAD v4.0 population databases (allele frequency=0) supports its classification as a rare variant rather than common polymorphism. Designated as a Tier 3 variant (uncertain clinical significance), potentially impairing DNA repair fidelity through indirect conformational effects rather than direct impairment of proofreading activity. This challenges the conventional paradigm linking POLD1 mutations exclusively to high-TMB

phenotypes, expanding our understanding of polymerase-related oncogenesis.

1.2 Immunotherapeutic Responsiveness in Low-TMB Context

Despite the patient's low tumor mutational burden (TMB), a remarkable response was observed to combined immunotherapy (PD-L1 inhibitor) and anti-angiogenic therapy (anti-VEGF), suggesting critical roles for TMB-independent mechanisms in modulating immune responses[2]. Current literature supports the hypothesis that tumor-specific genetic alterations may influence immune regulation. Richman et al. (2019) demonstrated that the immunogenicity of neoantigens correlates with their dissimilarity to self-proteins, implying that high-quality neoantigens—rather than quantity—may dominate therapeutic efficacy even in low-TMB contexts[3]. POLD1 mutation may enhance immunotherapy sensitivity by remodeling the tumor immune microenvironment (TIME), potentially through mechanisms such as cytokine signaling or antigen presentation rather than TMB-driven neoantigen production[4]. Furthermore, the synergy between anti-VEGF and immune checkpoint inhibitors might be amplified in low-TMB tumors. Fukumura et al. (2018) proposed that vascular normalization enhances T cell infiltration and functionality, an effect that varies across tumors with distinct mutational profiles[5]. Supporting this, post-treatment cytokine profiling in our patient showed pronounced Th1-polarized immune activation, corroborating the role of vascular remodeling in TIME modulation. These findings collectively highlight multidimensional crosstalk between genetic alterations, vascular dynamics, and immune reprogramming in low-TMB HCC.

S2. Multidimensional Mechanisms of Neurological Recovery

2.1 Critical Role of Acupuncture in Neural Restoration

From the perspective of local neuromodulation effects, the application of 5Hz continuous-wave electroacupuncture at L2-S1 Jiaji (EX-B2) points and lower extremity acupoints delivers low-frequency pulses that precisely target dorsal horn neurons of the spinal cord. This stimulation modulates ion channel permeability on neuronal membranes, promoting sodium influx to generate action potentials, thereby accelerating the recovery of motor nerve conduction[6]. After 4 weeks of treatment, the patient exhibited significant alleviation of lower limb sensory abnormalities, with electromyography (EMG) demonstrating marked improvements in nerve conduction velocities.These findings align with prior preclinical studies where electroacupuncture enhanced BDNF expression and axonal regeneration in spinal cord-injured rats[7].

At the systemic immunomodulatory level, electroacupuncture at Zusanli (ST36) and Weizhong (BL40) acupoints engages the vagus nerve-cholinergic pathway. As demonstrated by Tracey (2007)[8], this pathway triggers acetylcholine release to suppress inflammasome activation, thereby attenuating excessive production of pro-inflammatory cytokines like IL-6. Concurrently, electroacupuncture synergizes with immunotherapy to elevate IFN-γ and TNF-α levels, establishing a "pro-repair inflammatory microenvironment." Quantitatively, IL-6 levels decreased by 44% post-treatment, while IFN-γ surged to 96-fold above baseline. This

"neuro-immune crosstalk" phenomenon—though rarely documented in conventional rehabilitation—exhibits striking temporal alignment with both cytokine profile shifts and ASIA motor score improvements in our patient.

2.2 Temporal Correlation Between Cytokine Dynamics & Functional Recovery

In this case study, flow cytometry was used to systematically monitor multiple cytokines in the patient's serum to explore their temporal correlation with neurological recovery. The monitoring results showed that, three months after rehabilitation therapy, characteristic changes in cytokine levels were observed in the patient's body. Levels of interleukin-6 (IL-6), interleukin-8 (IL-8), interleukin-10 (IL-10), and interleukin-12 P70 (IL-12-P70) decreased, while interleukin-1β (IL-1β), interleukin-2 (IL-2), interleukin-4 (IL-4), interleukin-5 (IL-5), interleukin-17 (IL-17), tumor necrosis factor-α (TNF-α), interferon-γ (IFN-γ), and interferon-α (IFN-α) showed mild to moderate increases, with an increase of 1.5 to 3 times the baseline level. This pattern typically represents normal immune responses and repair processes in the body, and aligns with the patient's self-reported time of first being able to stand up.

Further analysis revealed that the changes in the patient's cytokine profile exhibited a balanced immune regulatory pattern, which is highly likely to be a key mechanism for promoting neurological recovery. At the cellular immune level, the CD4/CD8 ratio increased significantly from an initial 0.77 to 1.64, approaching the healthy range, indicating effective restoration of immune balance within the patient. Among these, the levels of IL-1β and TNF-α moderately increased, combined with the decrease in IL-6 and IL-8, creating a "controlled inflammation" environment. This environment

not only triggers necessary repair signals and initiates cell activities related to neural repair but also prevents excessive inflammatory responses from damaging the neural tissue[9].

From the perspective of the synergistic effect between immunotherapy and rehabilitation therapy, immunotherapy may participate in the neural repair process directly or indirectly by activating specific immune cell populations to release neuro-repair factors. Acupuncture and other rehabilitation therapies, on the other hand, may inhibit excessive inflammatory responses and regulate the neuro-immune interaction, thus creating a synergistic effect with immunotherapy. Together, they form a balanced state of cytokine networks, providing favorable conditions for the repair and functional recovery of damaged neural tissue.

It is worth noting that the slight decrease in anti-inflammatory factors such as IL-10 may reflect the body's transition from a high-anti-inflammatory state to a balanced immune state. In the early stages of disease, the body is in a stress state, with a large release of anti-inflammatory factors to reduce inflammatory damage; as rehabilitation progresses, there is a moderate reduction in anti-inflammatory factor levels, allowing for a moderate reparative inflammatory response, which is more beneficial for the repair and regeneration of neural tissue.

S3.Integrated Rehabilitation and Neurological Recovery (May 2023-Present)

The patient underwent a comprehensive rehabilitation protocol integrating advanced physical therapies and traditional Chinese medicine (TCM) interventions.

Modern physiotherapeutic strategies included hyperbaric oxygen therapy (2.0 ATA, 60 min/session), focused shockwave therapy (0.25 mJ/mm^2 energy flux density), polarized light irradiation (660 nm wavelength), low-frequency electrical bladder stimulation, neuromuscular electrical stimulation (NMES), and robotic-assisted gait training using lower-limb exoskeletons. These modalities collectively targeted muscle strengthening, neural pathway reactivation, and management of neurogenic bladder complications.

The TCM protocol centered on electroacupuncture (EA) with precise parameterization: G6805 stimulator delivering 5 Hz continuous bidirectional pulses (0.6 ms pulse width) at 5-8 mA intensity through 28-gauge stainless steel needles, administered daily for 30 minutes. Acupoint selection prioritized spinal cord injury segments and peripheral nerve regeneration zones. Primary stimulation sites included Jiaji points (EX-B2) located 1.25 cm lateral to the L2-S1 spinous processes (25-40 mm insertion depth) to modulate segmental spinal innervation; Zusanli (ST36) at 4 cm inferior to the Dubi point (ST35) with 25-40 mm depth to activate peroneal nerve branches; and Yaoyangguan (GV3) at the L4-L5 interspinous space (25 mm depth, 15° cephalad angulation) to enhance spinal vascular perfusion. Adjunctive acupoints such as Sanyinjiao (SP6) and Guanyuan (CV4) addressed urinary dysfunction, while Yanglingquan (GB34) and Chengshan (BL57) facilitated lower extremity strength recovery.

Reference

1. Chen, Huiqing et al. "Beyond proofreading: POLD1 mutations as dynamic orchestrators of genomic instability and immune evasion in cancer." Frontiers in immunology vol. 16 1600233.

2. Benmebarek MR, Karagiannis D, Chocarro de Erauso L, et al. Anti-vascular endothelial growth factor treatment potentiates immune checkpoint blockade through a BAFF- and IL-12-dependent reprogramming of the TME. Immunity. 2025;58:926-945.e10.

3. Richman LP, Vonderheide RH, Rech AJ. Neoantigen dissimilarity to the self-proteome predicts immunogenicity and response to immune checkpoint blockade. *Cell Syst* 2019;9:375-382.e4

4. Ma X, Dong L, Liu X, Ou K, Yang L. POLE/pold1 mutation and tumor immunotherapy. *J exp clin cancer res: CR* 2022;41:216

5. Fukumura D, Kloepper J, Amoozgar Z, Duda DG, Jain RK. Enhancing cancer immunotherapy using antiangiogenics: opportunities and challenges. *Nat Rev, Clin Oncol* 2018;15:325–40

6. Shin HK, Lee SW, Choi BT. Modulation of neurogenesis via neurotrophic factors in acupuncture treatments for neurological diseases. *Biochemical Pharmacology* 2017;141:132–42

7. Wang HP, Chen MR, He X, Chen ZW,Liang CH, Wang TH, et al. Electro-acupuncture promotes neuroplasticityassociated with systematic expressions of NTFs in spinal cord transected rats. *IntJ Clin Exp Med* 2017;10(2):2091-2103.

8. Tracey KJ. Physiology and immunology of the cholinergic antiinflammatory pathway. *J Clin Invest* 2007;117:289–96

9. Kelly MJ, Breathnach C, Tracey KJ, Donnelly SC. Manipulation of the inflammatory reflex as a therapeutic strategy. *Cell Rep Med* 2022;3:100696
